# Draft Genome Sequence of the Antimicrobial-Producing Strain Paenibacillus elgii AC13

**DOI:** 10.1128/genomeA.00573-18

**Published:** 2018-06-28

**Authors:** Daniel Barros Ortega, Rosiane Andrade Costa, Allan Silva Pires, Thiago Fellipe Araújo, Janaina Fernandez Araújo, Adriane Silva Kurokawa, Beatriz Simas Magalhães, Alessandra Maria Moreira Reis, Octávio Luís Franco, Ricardo Henrique Kruger, Georgios Joannis Pappas, Cristine Chaves Barreto

**Affiliations:** a Graduate Program in Genomic Sciences and Biotechnology, Universidade Católica de Brasília, Brasília, Distrito Federal, Brazil; b Graduate Program in Molecular Biology, Universidade de Brasília, Brasília, Distrito Federal, Brazil; c S-Inova Biotech, Biotechnology Postgraduate Program, Universidade Católica Dom Bosco, Campo Grande, Mato Grosso do Sul, Brazil; d Integra Bioprocessos e Análises, Brasília, Distrito Federal, Brazil

## Abstract

A Paenibacillus elgii strain isolated from soil samples from Cerrado, Brazil, showed antimicrobial activity. Its genome sequence was acquired (GS20 FLX Titanium 454 platform) and comprises 108 contigs (*N*_50_, 198,427 bp) and 6,810 predicted sequences. Here, we shed some light on the antimicrobial genes of the strain, including a nonribosomal peptide synthetase (NRPS) module identified as part of a pelgipeptin gene cluster.

## Abstract (precis)

A Paenibacillus elgii strain isolated from soil samples from Cerrado, Brazil, showed antimicrobial activity. Its genome sequence was acquired (GS20 FLX Titanium 454 platform) and comprises 108 contigs (*N*_50_, 198,427 bp) and 6,810 predicted sequences.

## GENOME ANNOUNCEMENT

The Paenibacillus genus was defined after an extensive comparison between DNA sequences encoding 16S rRNA (1). Since then, different species of this genus have been described as biological control agents and producers of molecules with surfactant properties and several lipopeptides with antimicrobial activity (2–4). Lipopeptides are cyclic or linear peptides carrying a variable fatty acid in their N-terminal region. These molecules are a product of nonribosomal synthesis. Differently from the ribosomal synthesis, nonribosomal peptide synthetases (NRPS) use a mixture of d- and l-amino acids and nonproteinogenic amino acids as building blocks (2).

Paenibacillus elgii strain AC13 is a spore-forming monoderm bacterium isolated from soil samples from the Brazilian Cerrado. This strain exhibits long-rod cells and motility by peritrichous flagella; growth occurs at pH values ranging from 4.7 to 8.0 at temperatures of 28°C and 37°C. Paenibacillus elgii strain AC13 also produces at least two groups of antimicrobial compounds (5–7).

The DNA of strain AC13 was isolated from an overnight culture on Luria-Bertani medium (37°C). Approximately 0.5 g of cells was collected, and DNA was extracted using a modified cetyltrimethylammonium bromide (CTAB) protocol (8). Cells were lysed using a solution containing 10% sodium dodecyl sulfate (SDS), 10% CTAB in 0.7 M NaCl, and 1 mg ml^−1^ lysozyme. Proteins were removed enzymatically with 100 ng ml^−1^ (wt/vol) proteinase K, followed by a phenol-chloroform-isoamyl alcohol (25:24:1 [vol/vol/vol]) extraction (9). The DNA was suspended in distilled water and quantified. The genome sequence was acquired using a GS20 FLX Titanium 454 platform with 20× coverage yielding 455,876 reads. The assembly was performed using the Newbler software (version 2.3) yielding 126 contigs, of which 108 contigs had more than 100 bp, with an *N*_50_ value of 198,427 bp, 53.4% G+C content, and 7,809,407 bp.

Genomic sequence annotation was performed using the Prokaryotic Genome Annotation Pipeline (PGAP), a modified NCBI Prokaryotic Genome Automatic Annotation Pipeline (PGAAP) protocol (10–16). The genome contained 7,149 total genes and 6,810 coding sequences (CDS), including 111 RNA genes, of which there were 7 5S rRNA genes, 7 partial 16S rRNA genes, 1 partial 23S rRNA gene, and 92 tRNA genes.

Forty putative NRPS genes were identified, and some of them might be part of the pelgipeptin synthase gene cluster that was identified in P. elgii strain B69 (17, 18). Other antimicrobial genes were identified, including a fusaricidin coding sequence, nine genes involved in polyketide gene cluster, eight genes related to bacteriocins, and genes for two ABC transporters related to lantibiotics.

The average nucleotide identity (ANI) comparison with other Paenibacillus genomes available at the NCBI genome database and the Joint Genome Institute (19) revealed ANI values of 70.23% with P. antarcticus CECT 5836, 70.3% with P. panacisoli DSM 21345, 70.46% with P. polymyxa CR1, 70.76% with P. crassostreae LPB0068, 70.78% with P. larvae BRL-230010, 70.95% with P. alvei TS-15, 74.96% with P. mucilaginosus K02, 96.45% with P. elgii B69, and 97.62% with P. elgii type strain M63, indicating that AC13 is a new Paenibacillus elgii strain.

### Accession number(s).

The Paenibacillus elgii strain AC13 whole-genome shotgun project sequencing has been deposited at DDBJ/ENA/GenBank under the accession number PYHP00000000. The version described in this paper is version PYHP01000000.
